# Characterisation and evaluation of predisposing factors for the development of xanthinuria in dogs with leishmaniosis under allopurinol therapy

**DOI:** 10.1186/s13071-025-06731-0

**Published:** 2025-03-10

**Authors:** Sara Clemente Oliveira, Carolina Arenas, Marina Domínguez-Ruiz, Eva Prosper, Maria Joana Dias, Rodolfo Oliveira Leal

**Affiliations:** 1 HEV–Hospital Escolar Veterinário, Faculdade de Medicina Veterinária, Universidade de Lisboa, Avenida da Universidade Técnica, 1300-477 Lisbon, Portugal; 2 Anicura Hospital Veterinario Valencia Sur, Avenida de Picassent, 28, 46460 Silla, Valencia Spain; 3 Hospital Clínico Veterinario, Universidad Alfonso X El Sabio, Avenida Universidad, 1, 28691 Villanueva de la Cañada, Madrid Spain; 4 Centro de Investigação Interdisciplinar em Sanidade Animal, Faculdade de Medicina Veterinária, ULisboa, Lisbon, Portugal; 5 Associate Laboratory for Animal and Veterinary Sciences (AL4AnimalS), Lisbon, Portugal

**Keywords:** Allopurinol, Canine leishmaniosis, Risk factors, Xanthinuria

## Abstract

**Background:**

Allopurinol, one of the drugs routinely used to treat canine leishmaniosis (CanL), is an inhibitor of the enzyme xanthine oxidase, which plays a fundamental role in purine metabolism. Its inhibitory action on this enzyme leads to a state of hyperxanthinuria, favouring the development of xanthine crystals and/or uroliths. However, not all dogs with CanL treated with allopurinol develop xanthinuria and/or xanthine uroliths, and there is not much information on the possible risk factors that contribute to this event. This study aims to evaluate potential predisposing factors associated with the development of xanthinuria in dogs with a previous diagnosis of CanL that were treated with allopurinol.

**Methods:**

A multicentric, retrospective, observational study was conducted and included dogs with CanL undergoing allopurinol therapy. Dogs that developed xanthinuria (Xgroup) and those without xanthinuria (NXgroup) were selected from cases admitted to three referral hospitals between 2011 and 2022. Medical records were reviewed, and clinical and laboratorial variables were compared between groups. Descriptive statistics, contingency tables and non-parametric tests were used (*P* < 0.05).

**Results:**

In total, 90 dogs were selected, 45 for each group. Only age and serum alpha-1 globulin concentration were significantly different between groups at day 0. Dogs from Xgroup were younger (median 4 years; interquartile range (IQR) 2–7) than those from NXgroup (median 6 years; IQR 4–9; *P* = 0.002). At the time of CanL diagnosis, a higher percentage of dogs from NXgroup had decreased serum alpha-1 globulin concentrations (38.9% versus 13.3% in Xgroup, respectively; *P* = 0.020). In Xgroup, the median time to xanthinuria development after starting allopurinol was 150 days (IQR 31–455).

**Conclusions:**

These results suggest that closer monitoring of young dogs (< 4 years) and those with normal alpha-1 globulin levels at diagnosis is recommended to ascertain the possible development of xanthinuria at an early stage, allowing for early application of measures to reduce the likelihood of its development.

**Graphical Abstract:**

**Figure:**
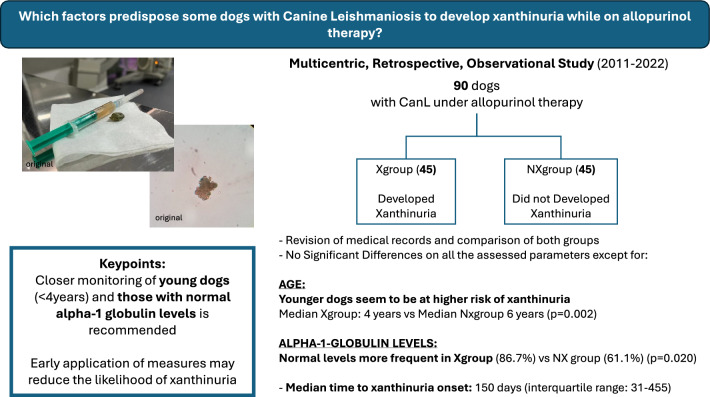

## Background

Canine leishmaniasis (CanL) is a chronic, multisystemic, insidious disease with a wide range of clinical presentations and degrees of severity that depend on various factors inherent to the parasite (*Leishmania infantum*), vector and vertebrate host [1]. Lesions and clinical signs manifest months to years after infection [2] and can be mostly non-specific, cutaneous, ocular or present in other forms such as lameness, epistaxis and neurological disorders [2, 3]. Frequent laboratory abnormalities include normocytic normochromic, non-regenerative anaemia, thrombocytopenia, hypergammaglobulinaemia, hypoalbuminaemia and proteinuria [2–4]. Diagnosis is based on an integrated approach that considers the history, clinical signs and laboratory abnormalities compatible with the disease and serological, molecular or cytologic diagnostic methods [3]. The choice of the most appropriate therapeutic protocol for CanL depends on the stage of the disease according to the international classification proposed by Leishvet [3]. The most widely used therapeutic protocol, and the one considered most effective, results from the combination of meglumine antimoniate and allopurinol [5–7] followed by long-term allopurinol use (maintenance treatment) [8, 9].

The development of xanthinuria is one of the potential side effects of allopurinol therapy [3]. In fact, allopurinol and its active metabolite (oxypurinol) inhibit the functioning of the enzyme xanthine oxidase (XO), an enzyme that plays a fundamental role in purine metabolism [10]. The inhibitory action of allopurinol leads to a decrease in the conversion of hypoxanthine into xanthine (a poorly water-soluble compound) and the latter into uric acid (a water-soluble compound), resulting in a state of hyperxanthinuria which favours the development of xanthine crystals and/or uroliths [11]. However, not all dogs with CanL treated with allopurinol develop xanthinuria and/or xanthine uroliths, and so far there is not much information on possible risk factors for this event. Therefore, the aims of this study were to describe the main characteristics of dogs diagnosed with CanL that were treated with allopurinol and developed xanthinuria as well as to assess the existence of potential risk factors for its development.

## Methods

### Data resource

A multicentric, retrospective, observational study was conducted which included dogs diagnosed with CanL between October 2011 and February 2022 that were treated with allopurinol in three veterinary referral hospitals (masked for review). A single database was created with information obtained from respective medical databases.

Whenever available, information collected included the following: time of diagnosis of CanL, age, sex, breed, reproductive status, body condition, weight, diet, serology titres, time of the year in which diagnosis was established, LeishVet clinical staging, clinical signs, systemic blood pressure values, clinicopathological findings (complete blood count, biochemical profile, electrolytes, serum protein electrophoresis, urine analysis, and urinary protein-to-creatinine ratio) at the time of CanL diagnosis, therapeutic approach undertaken and the date and method of diagnosis of xanthinuria.

### Inclusion criteria

Dogs were selected if they: (1) were diagnosed with CanL (natural infection by *L. infantum*) by obtaining a positive serological (quantitative methods – Leiscan® Leishmania ELISA Test) or molecular (quantitative real-time polymerase chain reaction (PCR)) result; (2) had clinical signs that justified allopurinol treatment; (3) did not have a previous history of xanthinuria known before allopurinol treatment; and (4) had available clinical information regarding time of diagnosis of CanL, age, sex, breed, reproductive status and weight, presence of xanthinuria and follow-up.

Dogs were divided into two groups: dogs that developed xanthinuria (Xgroup) and dogs that did not develop xanthinuria (NXgroup). To be included in the Xgroup, dogs may had been diagnosed with xanthinuria through the report of visualisation of xanthine crystals in urinalysis or detection of urolithiasis in abdominal ultrasound, followed by urinalysis and confirmation of the presence of xanthine crystals.

### Statistical analysis

The single database was created in Microsoft® Office Excel 2013. Statistical analysis was performed using IBM® SPSS® Statistics (SPSS version 23.0) and Rx64 (version 4.2.2). For qualitative variables, the existence of risk factors for the development of xanthinuria was assessed using Pearson’s chi-squared test or the Fisher’s exact test, with a confidence interval of 95% (*P* = 0.05). Statistical significance was set at *P* < 0.05. For quantitative variables, the Shapiro–Wilk test was carried out to test normality. The existence of statistical associations with medians was assessed using the Wilcoxon rank-sum test (non-parametric test). Data were presented as medians and their interquartile ranges (25–75th).

## Results

In total, 90 dogs met the inclusion criteria, consisting of 45 dogs in each group (Xgroup versus NXgroup).

### Temporal distribution of CanL diagnosis

In Xgroup, the diagnosis of CanL was made mainly in December, 13.3% (6/45); May, 11.1% (5/45); July, 11.1% (5/45); and August, 11.1% (5/45) and in theNXgroup, in the months of November, 16.7% (7/42); March, 14.3% (6/42); February, 11.9% (5/42); and May, 11.9% (5/42). Three dogs in NXgroup only had information regarding the year of CanL diagnosis.

### Age, sex, reproductive status and breed

**Fig. 1:**
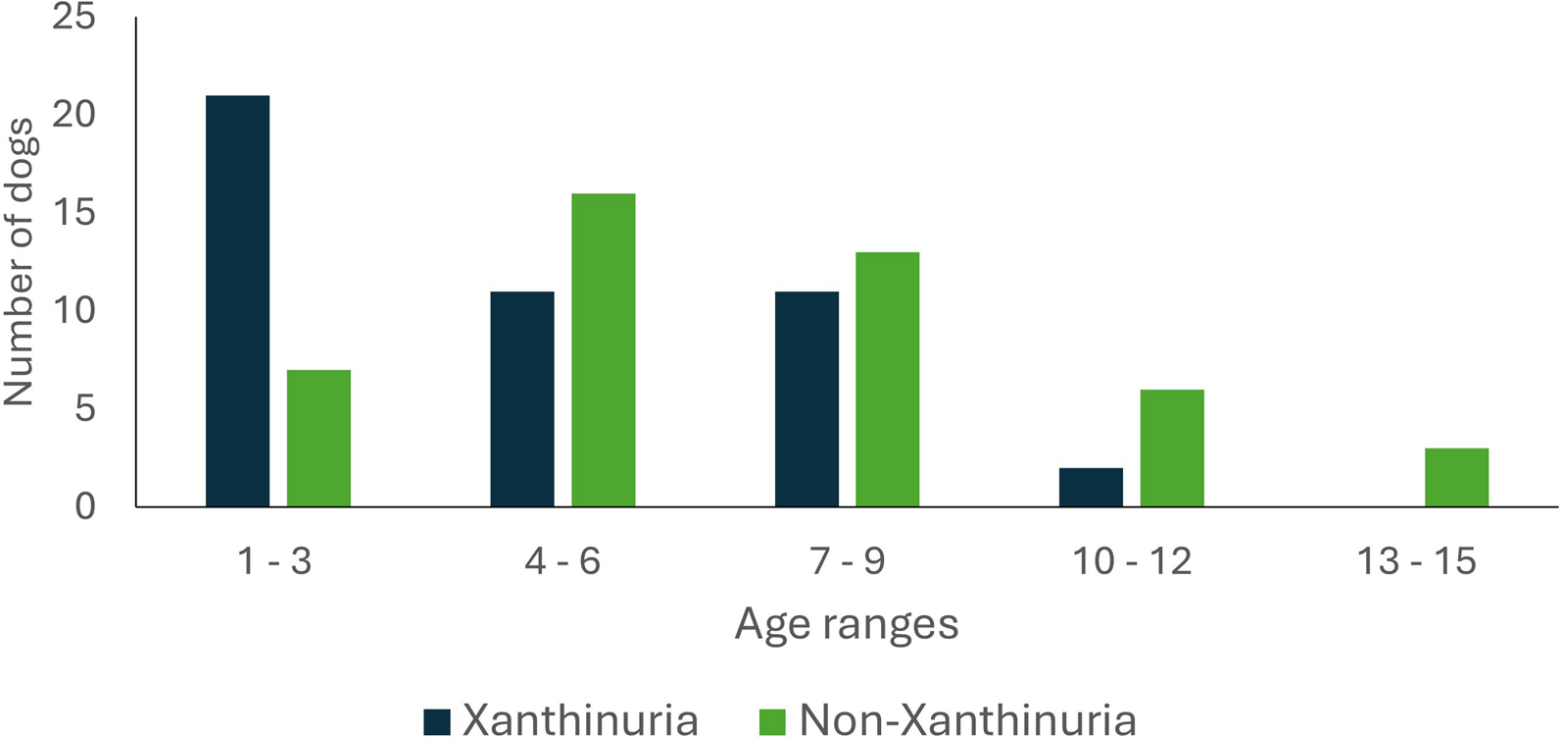
Age range distribution of dogs with CanL treated with allopurinol that developed and did not develop xanthinuria (Xgroup and NXgroup, respectively)

The median age of Xgroup was 4 years (IQR: 2–7). In detail, 46.7% (21/45) of the dogs were between 1 and 3 years old. In NXgroup, the median age was 6 years (IQR: 4–9), where 35.6% (16/45) were between 4 and 6 years old and 28.9% (13/45) between 7 and 9 years old (Fig. 1). In this study, age was statistically different (*P* = 0.002) between groups, with animals in Xgroup being younger. Dogs that were 4 years old or younger had 3.14 times greater odds of developing xanthinuria compared with older ones.

In addition, sex and reproductive status were not statistically different between groups (*P* = 1 and *P* = 0.970, respectively). Both groups were composed of 29 males (64.4%) and 16 females (35.6%). A total of 55.6% (25/45) and 57.8% (26/45) of the dogs were intact in Xgroup and NXgroup, respectively.

Xgroup had 32 purebred dogs (71.1%). Of the 24 breeds represented, the most prevalent were: Boxer, 9.4% (3/32); Labrador Retriever, 9.4% (3/32); and German Shepherd, 9.4% (3/32). NXgroup had 31 purebred dogs (68.9%). Of the 25 breeds present, German Shepherd, 9.7% (3/31), was the most prevalent, followed by the American Pit Bull Terrier, 6.5% (2/31); Labrador Retriever, 6.5% (2/31); English Pointer, 6.5% (2/31); and English Setter, 6.5% (2/31). Regarding breed, there were no statistically significant differences between the groups (*P* = 0.820).

### Body condition, weight and diet

The median weight was 22.5 kg (IQR 13.2–32.8) for Xgroup and 20.0 kg (IQR 11.1–28.5) for NXgroup. In Xgroup, body condition was only available in 51.1% (23/45) of the dogs of which 65.2% (15/23) had an ideal body condition (4–5). Regarding NXgroup, it was possible to obtain this information in 42.2% (19/45) of the cases of which 57.9% (11/19) had an ideal body condition (4–5). The median value for body condition was 5 (IQR 3–5) in Xgroup and 4 (IQR 3–5) in NXgroup. There were no significant differences between groups in terms of weight (*P* = 0.534) and body condition (*P* = 0.466).

Diet history before CanL diagnosis was recorded in 57.8% (26/45) and 64.4% (29/45) of dogs in Xgroup and NXgroup, respectively. A total of 92.3% (24/26) and 75.9% (22/29) of dogs in Xgroup and NXgroup, respectively, were eating commercial dry food. In Xgroup, at the time of CanL diagnosis, the diet was changed in 23.7% (9/38) of the cases and from these, 55.6% (5/9) switched to a low-purine diet. In NXgroup, there was a change in diet in 15.6% (7/45) of the cases of which 28.6% (2/7) switched to a low-purine diet. There were no significant differences regarding diet change at the time of CanL diagnosis (*P* = 0.350) and eating a low-purine-content diet (*P* = 0.237).

### Serology and LeishVet clinical staging

A high positive serology was most prevalent in dogs from both groups (Xgroup, 54.8% (23/42); NXgroup, 46.5% (20/43)). There were no significant differences between groups regarding serology values (*P* = 0.293). Concerning LeishVet clinical staging, in both groups, stage II was the most frequent (Xgroup, 75.0% (33/44); NXgroup, 83.7% (36/43)), being similar in both groups (*P* = 0.852).

### Clinical signs

Non-specific and cutaneous clinical signs were the most prevalent in both groups. In five dogs, three from Xgroup and two from NXgroup, physical examination was considered normal.

### Complete blood count (CBC)

Regarding the CBC and biochemical profile, anaemia (53.7% versus 47.7%); thrombocytopenia (29.3% versus 25.0%); hypoalbuminaemia (22.5% versus 25.0%); hyperglobulinaemia (22.5% versus 13.6%); and hyperproteinaemia (20.0% versus 22.7%) were the most frequent findings in both groups. None of the abnormalities found in the CBC and biochemical profile were statistically different between groups.

### Biochemical profile and serum protein electrophoresis

**Fig. 2:**
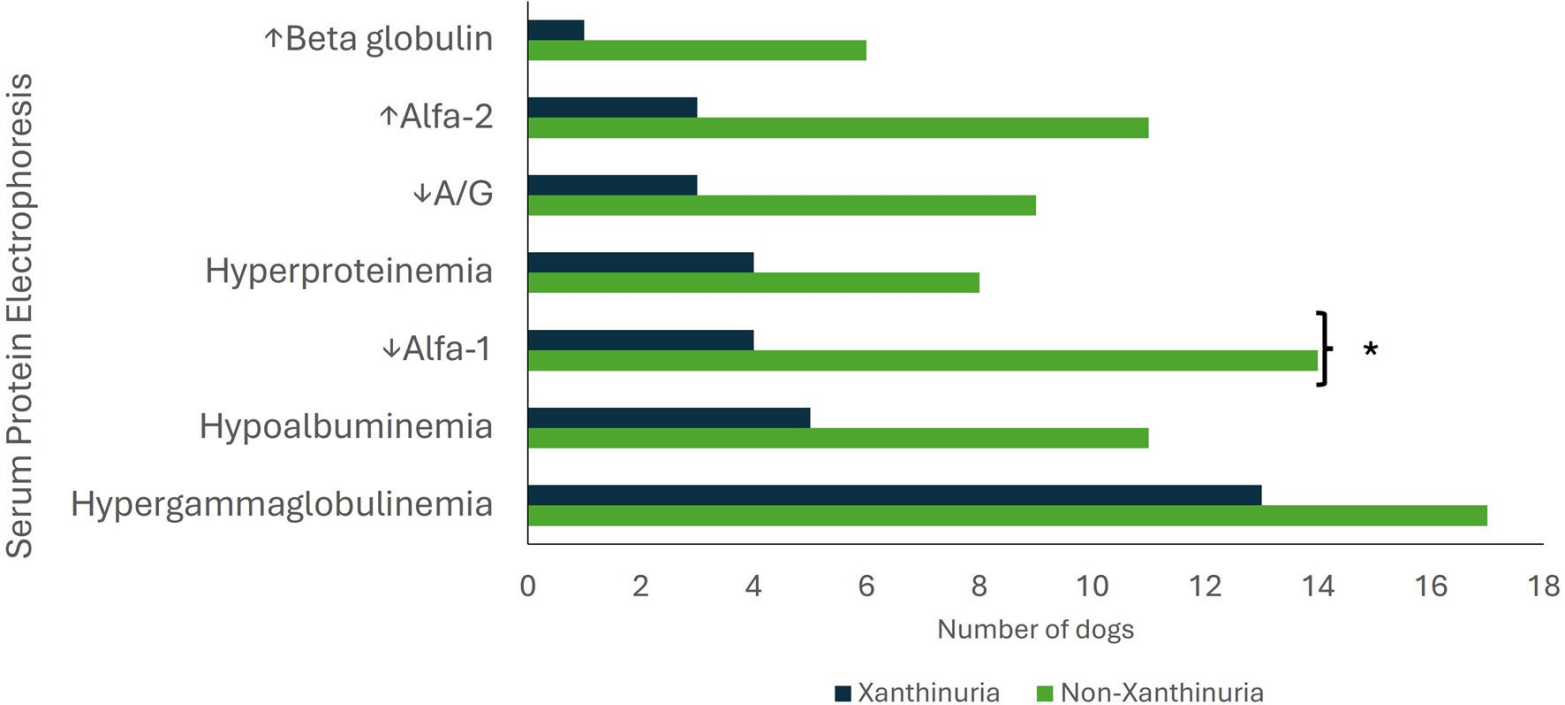
Serum protein electrophoresis changes identified at the time of diagnosis of CanLeish in dogs that later developed xanthinuria (Xgroup) or did not develop xanthinuria (NXgroup) under allopurinol treatment. The asterisk refers to statistically significant differences observed between groups (chi-square test; *P* = 0.02)

In serum protein electrophoresis, the presence of hypergammaglobulinaemia was the most prevalent finding in both groups (Xgroup, 43.3%; NXgroup, 47.2%). Xgroup also presented with hypoalbuminaemia (16.7%), a decrease in alpha-1 globulins (13.3%), and hyperproteinaemia (13.3%) and NXgroup showed a decrease in alpha-1 globulin concentration (38.9%), hypoalbuminaemia (30.6%) and an increase in alpha-2 globulin concentration (30.6%) (Fig. 2). Only the decrease in alpha-1 globulin concentration was significantly different between the groups (*P* = 0.020). The odds of having normal (instead of decreased) alpha-1-globulins were 4.3 times higher in Xgroup compared with NXgroup (odds ratio 4.3).

### Urine analysis and urinary protein-to-creatinine (UPC) ratio

The UPC ratio was available in 73.3% (33/45) and 66.7% (30/45) of dogs from Xgroup and NXgroup, respectively. A total of 48.5% (16/33) and 53.3% (16/30) of dogs, respectively, showed significant proteinuria. There were no significant differences regarding UPC ratio values between the groups (*P* = 0.938).

Urine analysis was available in 29 dogs (64.4%) from Xgroup. A total of 72.7% of dogs in Xgroup had a normal urinary pH (5.5 ≤ pH ≤ 7.5) and in 34.5%, the urine was hypersthenuric (urine-specific gravity (USG) ≥ 1.050). Proteinuria was found in 79.3% of the cases. In NXgroup, 73.3% and 40.0% of the dogs had pH and urine-specific gravity values within the reference ranges, respectively (5.5 ≤ pH ≤ 7.5; 1.035 ≤ USG < 1.050). Proteinuria was identified on dipstick in 73.3% of the cases, where information regarding urinalysis was available (66.7% (30/45)). None of the abnormalities found were statistically different between groups.

### Treatment

Around 33.0% (15/45) and 46.7% (21/45) of the dogs from Xgroup and NXgroup, respectively, received an immunomodulatory drug as part of the therapeutic approach. There were no differences between groups regarding the use of an immunomodulator (*P* = 0.197), nor between the various immunomodulators used (Leisguard®, Impromune® or Omnifect® Pro imune) (*P* = 0.054).

The median duration of allopurinol treatment was 150 days (IQR: 31–455) in Xgroup and 284 (IQR: 180–435) in NXgroup. In both groups, the most commonly used protocol was 10 mg/kg twice a day (BID) (Xgroup , 81.4%; NXgroup , 86.7%). In addition, 25.0% and 37.8% of Xgroup and NXgroup dogs, respectively, received allopurinol for more than 365 days. The allopurinol protocol (*P* = 0.598) and the use of this drug for more than 365 days (*P* = 0.194) were not statistically different between the groups. The duration of allopurinol treatment was statistically different (*P* = 0.005).

A total of 36 (80.0%) and 38 dogs (84.4%) from Xgroup and NXgroup, respectively, were treated with meglumine antimoniate (Glucantime®) in combination with allopurinol. In both groups, the most commonly used protocol for meglumine antimoniate was 50 mg/kg BID (Xgroup, 58.8%; NXgroup, 57.9%). The meglumine antimoniate protocol was not statistically different between the groups (*P* = 0.364).

### Xgroup – xanthinuria

Development of xanthinuria was seen between 4 and 2190 days after the beginning of allopurinol treatment. Xanthinuria diagnosis in the Xgroup dogs was more frequent in October (17.8% (8/45)), February (13.3% (6/45)) and January (11.1% (5/45)). The xanthinuria diagnosis was made through the visualisation of xanthine crystals in the urinalysis in 80.0% (36/45) of the cases and in the remaining 20.0% (9/45) through abdominal ultrasound, followed by urinalysis. In dogs with available information, the urinary pH value was found to be within the reference range (5.5 ≤ pH ≤ 7.5) in 71.0% (22/31) of the cases, while it was  ≥ 7 in 67.7% (21/31) of the dogs. USG was ≥ 1.035 in 62.2% (23/37) of the dogs. Proteinuria was identified in 57.5% (23/40) of the dogs.

## Discussion

This study assessed the existence of risk factors for the development of xanthinuria in dogs with CanL treated with allopurinol, allowing for the characterisation of two dog groups, one with and one without xanthinuria development.

In our study, there were no statistically significant differences regarding sex and breed. However, both xanthine urolith development and CanL diagnosis are more frequently documented in purebred dogs [11–18] and in males [11–20]. The number of males and females was equal between groups, with a predominance of males, as described in previous studies [21–23]. In both groups, the presence of purebred dogs was higher, as seen in other studies [17, 18, 21, 22], with the most frequent breeds being Boxer, Labrador Retriever and German Shepherd. These results are in line with what has been described regarding the greater susceptibility of some breeds to developing CanL owing to their genetic pool [24–26].

Reproductive status was not statistically different either. However, although the number of neutered females was higher than intact ones, this was not the case for males, with a higher percentage of intact males in both groups. Curi et al. [27] described that the prevalence of CanL was statistically higher in neutered dogs, although there are other studies in which the prevalence of intact dogs, although not evaluated in statistical terms, was higher [22]. The presence of xanthine uroliths is more frequently described in both sexes in sterilised animals [11, 15, 16, 19, 20]. However, to our knowledge, the study conducted by Torres et al. [22] was the only one in which a brief characterisation was made of the 13.0% of dogs (42/320) that, after being diagnosed with CanL and starting treatment, actually developed xanthinuria (and not xanthine uroliths), the number of intact males with xanthinuria being higher than that of castrated dogs.

In the present study, age was significantly different between groups, with dogs from Xgroup being younger. In this study, the pattern found in Xgroup (higher prevalence of CanL in the 1–3-year-old group) is similar to that described by Gálvez et al. [28] in which the presence of CanL was significantly higher in young dogs (< 1 year old). The pattern of age distribution in NXgroup (higher prevalence of CanL in the 4–6- and 7–9-year-old groups) was similar to that reported by Velez et al. [29], who described a bimodal distribution where there were two peaks, the first between the ages of 3 and 4 years and the second between the ages of 7 and 8 years. The presence of xanthine uroliths was most frequently documented in dogs aged between 4 and 7 years and 10 and 15 years [11, 15, 16, 19]. In the study conducted by Torres et al. [22], the median age at the time of CanL diagnosis of the 42 dogs with CanL that were treated with allopurinol and developed xanthinuria was provided but not the median age of the 278 dogs with CanL that were treated with allopurinol and did not develop xanthinuria. Therefore, to the author’s knowledge, there is no previous study comparing the ages of dogs with and without xanthinuria, both with CanL, at the time of diagnosis of CanL. According to the available literature, some studies point to an increase in XO activity with age in various tissues [30–32]. Vida et al. [31] analysed the activity and expression of XO in the liver, kidneys, thymus and plasma in rats with different ages (28, 52, 72 and 122 weeks). According to the obtained results, both parameters increased with age. Levinson and Chalker [33] found a correlation between XO activity and uric acid formation detailing that rats with higher XO activity exhibited greater uric acid excretion. On the basis of these previous studies, the fact that dogs in Xgroup were significantly younger may be attributed to the possible existence of lower XO enzyme activity and expression at the hepatic level in younger age. We hypothesised that in a young dog (with a lower quantity and activity of the enzyme) a more extensive inhibition of purine metabolism at the steps catabolized by XO (conversion of hypoxanthine to xanthine and then to uric acid) would be expected for the same dose of allopurinol compared with an adult dog (with a higher quantity of XO and greater activity). This inhibition would result in a higher urinary excretion of xanthine and hypoxanthine, thus facilitating the development of xanthinuria in these dogs. However, to the best of our knowledge, the assessment of XO activity and expression at the hepatic level in animals of various ages has not been evaluated in dogs, with available studies being conducted in rats. Therefore, further studies are needed to assess how reasonable this theory is and to justify the data obtained in the present study.

Regarding CBC and biochemical profile, none of the evaluated parameters showed statistically significant differences between the groups, indicating that no clinicopathological findings are predictors of xanthinuria. The presence of anaemia and thrombocytopenia were the most frequent findings observed, as described in other studies [23, 34].

In serum protein electrophoresis, the decrease in alpha-1 globulin concentration was statistically different between groups, with dogs in NXgroup more frequently showing a decrease in the concentration of these globulins. The presence of hypergammaglobulinaemia was the most frequent alteration in both groups, in line with literature [4, 35]. In clinical practice, serum protein electrophoresis is often used as an auxiliary diagnostic tool, allowing for the identification of inflammation and changes in serum protein fractions, which typically occur in certain diseases such as CanL, feline infectious peritonitis or ehrlichiosis [36]. In our study, dogs from NXgroup more often showed a decrease in serum alpha-1 globulin concentration. Since literature is scarce about this finding, the authors hypothesize several potential explanations. The first one is that, similarly to previous studies, this abnormality may be merely an incidental finding, without clinical significance [37, 38]. Secondly, the result could be a false positive, meaning that despite being statistically different, it may not be significant (type I error). At a significance level of 0.05, there is a 5% probability of committing a type I error [39]. Lastly, the third hypothesis is linked to a potential alteration in allopurinol pharmacokinetics. After entering the bloodstream, drugs circulate through the vascular compartment either freely or bound to proteins or blood cells. The main transport proteins include albumin, orosomucoid and lipoproteins [40–42]. Orosomucoid has only one drug-binding site and a much lower plasma concentration than albumin. Therefore, for substances with a higher affinity to this protein, their free fraction will be significantly altered if there is a variation in orosomucoid concentration [40, 41]. According to studies conducted in humans, both allopurinol and its active metabolite bind poorly to plasma proteins [43–45]. However, in NXgroup, a more frequent decrease in alpha-1 globulin concentration, to which orosomucoid belongs, was observed compared with Xgroup. Therefore, the suggested hypothesis would be that in Xgroup, where this fraction was mostly within reference values, there would be a greater binding of allopurinol to these proteins compared with NXgroup, leading to a prolongation of the drug’s action, favouring the inhibition of the XO enzyme and the development of xanthinuria. Further studies evaluating allopurinol pharmacokinetics concerning its potential binding to orosomucoid and the effects on the plasma concentration of this drug with variations in orosomucoid concentration are needed to support/refute this hypothesis.

The duration of allopurinol treatment was statistically different between groups (*P* = 0.005). This difference may be explained by the inclusion criteria used in NXgroup. It could not be guaranteed that dogs in NXgroup would not develop xanthinuria during some period of their lives while being treated with allopurinol. However, to try to minimize this probability, only dogs with CanL treated with allopurinol for a minimum period of 5 months were included in NXgroup, a value corresponding to the median (150 days) of allopurinol treatment and the onset of xanthinuria in Xgroup, with no record of xanthinuria development in clinical records during the entire monitoring period of the case. In our study, 81.4% of dogs that developed xanthinuria followed the recommended dose of 10 mg/kg BID [3]. The LeishVet group recommends the use of allopurinol for a minimum period of 6–12 months [3]. In this study, 25.0% and 37.8% of dogs in Xgroup and NXgroup, respectively, took allopurinol for more than 365 days, with these values also not being statistically different. Thus, despite the small sample size, in our study, the allopurinol dosage used did not appear to influence the development of xanthinuria.

In this study, the onset of xanthinuria occurred between 4 and 2190 days after the initiation of allopurinol treatment. Torres et al. [22] documented the development of xanthinuria, renal mineralization and/or urolithiasis between 3 weeks and 9 years (21 to 3285 days) after allopurinol treatment onset. Torres et al. [46] described the development of xanthine stones after more than 24 months of allopurinol therapy. However, although it was mentioned that the follow-up of the dogs included in the study (general health examination and complementary tests, including urinalysis) occurred 1, 3 and 6 months after CanL diagnosis and every 6 months thereafter, little information is available about the results obtained in these exams. In addition, there is no information on the presence of xanthinuria in the urine analyses performed, with only the development of xanthine uroliths in three dogs being described. Segarra et al. [47] reported the onset of xanthinuria in the group of dogs with CanL that received allopurinol, 30–180 days after the start of treatment. However, this study lasted only 180 days, and clinical evaluation and complementary tests (including urinalysis) were only performed on the day the animals entered the study (day 0) and after 30 and 180 days of treatment. In our study, the dog that developed xanthinuria 4 days after the initiation of allopurinol treatment was a 3-year-old, intact male Bulgarian Shepherd. As a retrospective study, it was not possible to assess the presence of other factors that could contribute to the development of xanthinuria before allopurinol treatment, such as impaired liver function. Similarly, it was not possible in this case to perform urinalysis before CanL diagnosis to investigate the potential presence of xanthinuria (hereditary xanthinuria). To the author’s knowledge, there is no reported predisposition of this breed to the development of xanthine crystals and/or uroliths. However, the available literature on diseases for which the Bulgarian Shepherd dog is more predisposed is limited, so an underlying genetic origin cannot be ruled out. Nevertheless, the allopurinol dose received by this animal was the recommended dose by the LeishVet group (10 mg/kg BID) [3], so we cannot find a plausible justification for such an early onset of xanthinuria in this dog.

Xanthine crystals were observed at urinary pH values below and above 7 (pH < 7 in 32.3% and pH ≥ 7 in 67.7%), which aligns with the relative insolubility of xanthine as described in literature, regardless of urinary pH [10, 11, 19, 48, 49].

This work constitutes a multicentric, retrospective, observational study that presents some limitations, namely: the occasional unavailability of information necessary to fill in the evaluated parameters; the fact that not all laboratory analyses were carried out by the same laboratories, which complicates the standardisation of the results; regarding the selection of NXgroup, although three of the inclusion criteria were “allopurinol treatment after CanL diagnosis for at least 5 months”, “no previous history of xanthinuria development” and “no development of xanthinuria during the clinical follow-up of the animal”, it cannot be guaranteed that the dogs included in NXgroup, after the follow-up period of this study, would not develop xanthinuria at some point.

## Conclusions

Considering our results, dogs with CanL diagnosis at a younger age and without a decrease in the concentration of alpha-1 globulins in the proteinogram performed at the time of diagnosis of this disease appear to be more prone to develop xanthinuria after allopurinol treatment. These results suggest that closer monitoring of dogs with these characteristics should be instituted to ascertain the possible development of xanthinuria at an early stage and allow the application of measures to reduce the likelihood of its development.

## Data Availability

No datasets were generated or analysed during the current study.

## Abbreviations

BID: Twice a day
CanL: Canine leishmaniosis
PCR: Polymerase chain reaction
UPC ratio: Urinary protein-to-creatinine ratio
USG: Urine-specific gravity
XO: Xanthine oxidase
